# Books and Magazines

**Published:** 1902-03

**Authors:** 


THE STANDARD MEDICAL DIRECTORY OF NORTH AMERICA, consisting of twelve parts, including Directory of Physicians of North America, Medical Colleges, Medical Service of the United States, Medical Societies, Medical Practice Acts, Medical Publications (including Books and Periodicals), Mineral Springs, Drugs and Medicines, Medical and Surgical Products, Manufacturers and Life Insurance Companies. Handsomely bound in red buckram, 824 pages, imperial octavo. Price, $10.00. G. P. Engelhard & Co., Chicago.

This work is a distinct improvement over any similar publication, in scope, accuracy and typography. It is not too bulky to handle. It is divided into twelve parts, each complete in itself, thereby facilitating reference and exhibiting in a concise form the extent and importance of the interests represented by the respective parts. It is a handsome volume, and will be "ornamental as well as useful."

Dr. Lott's Paper on "The Drug Habit: Its Treatment," was published in this journal for November, 1901. It was read at the Calvert meeting of the Brazos Valley Association. Since its publication Dr. Lott has received so many letters from physicians asking if he would receive such cases for treatment that he takes this method of announcing that he is prepared to and will receive and treat cases of opium, cocaine, chloral or whiskey addiction by the hyoscine treatment as described in the paper referred to. The method is well known to the readers of this journal. It is speedy and painless. Physicians who do not care to treat these cases, or who are not prepared to do so, are invited to correspond with him, and can send them to him with every assurance that they will be in good hands and will be treated strictly in accordance with ethical principles. They will be cared for in a private family residence, where Dr. Lott will personally treat them, and will be in the charge of skilled, trained and experienced nurses. Every comfort and convenience will be furnished, in the privacy of a home (not a sanitarium), including good family fare and cooking; privacy, baths, etc. Ladies will be received as well as men, and will have the services and constant attention of trained lady-nurses. Dr. Lott refers by permission to the editor of the Texas Medical Journal. Address Dr. M. K. Lott, Cameron, Texas.

